# Cost-effectiveness analysis of olanzapine in four-drug antiemetic therapy in Japanese patients treated with highly emetogenic cisplatin-containing chemotherapy

**DOI:** 10.1186/s40780-022-00246-x

**Published:** 2022-06-01

**Authors:** Yu Kondo, Tomoya Tachi, Takayoshi Sakakibara, Jun Kato, Takahito Mizuno, Yoshio Miyake, Hitomi Teramachi

**Affiliations:** 1 Department of Pharmacy, Toyota Kosei Hospital, 500-1, Ibobara, Jousui-cho, Toyota, 470-0396 Japan; 2 Laboratory of Clinical Pharmacy, Gifu Pharmaceutical University, Daigaku-nishi 1-25-4, Gifu, 501–1196 Japan

**Keywords:** Olanzapine, Cost-effectiveness, Highly emetogenic chemotherapy, Cost-utility analysis, Chemotherapy-induced nausea and vomiting

## Abstract

**Background:**

Olanzapine has been shown to have an additive effect on the three-drug antiemetic therapy consisting of aprepitant, palonosetron, and dexamethasone, in a highly emetogenic cisplatin-containing chemotherapy. Although olanzapine may be more economical than aprepitant or palonosetron, an adequate cost-efficacy analysis has not been conducted.

**Methods:**

We conducted a cost-utility analysis to evaluate the cost-effectiveness of olanzapine use in four-drug antiemetic therapy among Japanese patients. We simulated model patients treated with highly emetogenic cisplatin-containing chemotherapy and developed a decision-analytical model of patients receiving triple antiemetic therapy with or without olanzapine in an inpatient setting. The cost and probabilities of each treatment were calculated from the perspective of the Japanese healthcare payer. The probabilities, utility value, and other costs were obtained from published sources. One-way and probabilistic sensitivity analyses were conducted to examine the influence of each parameter on the model and the robustness of a base-case analysis. Threshold analysis was conducted to determine the cost of olanzapine that would make the incremental cost-effectiveness ratio (ICER) equivalent to the threshold ICER). The threshold incremental cost-effectiveness ratio was set at 5 million Japanese Yen (JPY) per quality-adjusted life-year (QALY) gained.

**Results:**

The cost was 10,238 JPY in the olanzapine regimen and 9719 JPY in the non-olanzapine regimen. The QALY gained were 0.01065 QALYs and 0.01029 QALYs in the olanzapine and non-olanzapine regimen, respectively. The incremental cost of the olanzapine regimen relative to the non-olanzapine regimen was 519 JPY, and the incremental QALYs were 0.00036 QALY, resulting in an ICER of 1,428,675 JPY per QALY gained. In the one-way sensitivity analysis, the results were most sensitive to the utility value of incomplete control. The probabilistic sensitivity analysis revealed the probability that the ICER was below the willingness-to-pay, and the incremental QALYs was positive was 96.2%. The calculated cost of olanzapine per 5 mg that would make the incremental cost-effectiveness ratio equivalent to the threshold incremental cost-effectiveness ratio was calculated to be 475 JPY.

**Conclusions:**

Olanzapine was cost-effective in the four-drug antiemetic therapy for Japanese patients treated with highly emetogenic cisplatin-containing chemotherapy.

## Background

Nausea and vomiting decrease the quality of life (QOL) of patients undergoing cancer chemotherapy [1]. The control of nausea and vomiting is significant for the continuation of treatment. The guidelines of scientific societies, such as the American Society of Clinical Oncology (ASCO) [2], the Multinational Association of Supportive Care in Cancer/European Society of Medical Oncology (MASCC/ESMO) [3], and the National Comprehensive Cancer Network (NCCN) [4], classify the emetogenicity of anticancer drugs into four levels. Similarly, the Japan Society of Clinical Oncology guideline classifies emetogenicity into minimal (< 10%), low (10–30%), moderate (30–90%), and highly emetogenic (> 90%) risks [5]. Cisplatin is one of the most emetogenic anticancer drugs, and all guidelines have classified cisplatin as highly emetogenic. Adequate prophylactic antiemetic therapy is essential to maintain the QOL of patients taking highly emetogenic regimens.

Olanzapine inhibits multiple receptors (dopamine D_1_, D_2_, D_3_ receptors, serotonin 5-hydroxytryptamine type 2a (5-HT_2a_), 5-HT type 2c (5-HT_2c_), 5-HT_3_, and 5-HT_6_ receptors, alpha1-adrenergic receptors, muscarinic receptors, and histamine H_1_ receptors) [6]. Olanzapine has been used to treat schizophrenia, but it has recently emerged as an antiemetic agent in cancer chemotherapy [7–11]. A randomized, double-blind, placebo-controlled phase III study reported that adding olanzapine to neurokinin-1 (NK_1_) receptor antagonists, 5-HT_3_ receptor antagonists, and dexamethasone brought significant benefits in preventing chemotherapy-induced nausea and vomiting (CINV) from highly emetogenic cisplatin-containing chemotherapy [8]. The ASCO guidelines recommended a four-drug combination, including a NK_1_-receptor antagonist, 5-HT_3_ receptor antagonist, dexamethasone, and olanzapine, for preventing CINV after highly emetogenic chemotherapy (HEC) [2]. One of the recommended options for use in the NCCN and MASCC/ESMO guidelines was a four-drug combination that included olanzapine [3, 4].

Japan has a universal health insurance system, and the Ministry of Health, Labor, and Welfare determines drug prices. The rapid aging of Japan’s population and the resulting increase in medical costs have become a severe problem. Because NK_1_ receptor antagonists, such as aprepitant and 5-HT_3_ receptor antagonists, are more expensive than classical antiemetics, several studies have examined their cost-effectiveness in Japan and overseas [12–16]. In Japan, aprepitant use was reportedly cost-effective for outpatient treatment but not cost-effective under inpatient conditions [16]. Palonosetron, a second-generation 5-HT_3_ receptor antagonist, was reportedly not cost-effective as a first-generation 5-HT_3_ receptor antagonist [14].

On the other hand, the drug price of olanzapine (branded) per 5 mg of is 150.4 Japanese yen (JPY) (1.41 United States dollars (USD)) (generic, 28.9 JPY (0.27 USD), which is lower than those of aprepitant (branded, 8949.3 JPY (83.81 USD)/3 days); generic, 3904.4 JPY (36.61 USD)/3 days) and palonosetron (branded, 14,937 JPY (138.27USD); generic, 5349 JPY (50.10 USD)). Since the price of olanzapine is low and has shown sufficient antiemetic effect in the J-FORCE study, it may be recommended from the perspective of cost-effectiveness. However, no quantitative cost-effectiveness analysis of olanzapine has been conducted for the four-drug combination. Although there have been overseas reports examining the cost-effectiveness of olanzapine in HEC [17, 18], none of them have examined the cost-effectiveness of adding olanzapine to the three-drug combination. In addition, it is difficult to extrapolate the results of overseas studies directly to Japan because of the differences in medical costs and insurance systems between Japan and other countries. Given this, this study aimed to evaluate the cost-effectiveness of olanzapine quantitatively in four-drug antiemetic therapy among Japanese patients receiving highly emetogenic cisplatin-containing chemotherapy.

## Methods

### Model

**Fig. 1:**
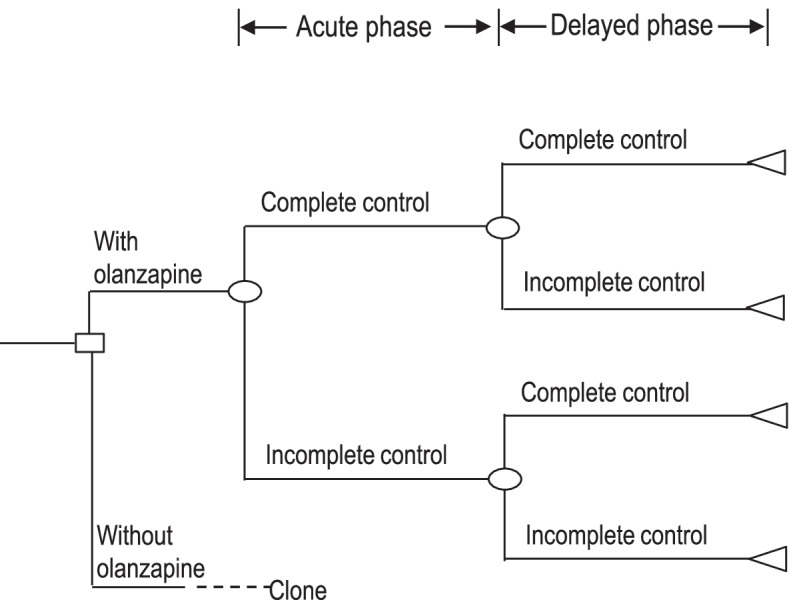
Decision-analytic model for cost-utility analysis. The decision tree shows the four possible health states that a model patient can experience after receiving an antiemetic regimen. Olanzapine-containing regimen comprised dexamethasone, palonosetron, aprepitant, and olanzapine

We simulated model patients treated with highly emetogenic cisplatin-containing chemotherapy and developed a decision-analytic model (Fig. 1). Based on a phase III clinical trial conducted in Japan (J-FORCE study) [8], the model patients received either a four-drug (olanzapine regimen) or three-drug regimen (non-olanzapine regimen) in an inpatient setting. In the J-FORCE study, key inclusion criteria were treatment with cisplatin (≥50 mg/m^2^) for the first time, age between 20 and 75 years, and with an Eastern Cooperative Oncology Group performance status (ECOG-PS) of 0–2. A total of 710 patients were assigned to either the olanzapine regimen (356 patients) or non-olanzapine regimen (354 patients). The olanzapine regimen included dexamethasone (12 mg on Day 1 and 8 mg on Days 2–4), palonosetron (0.75 mg on Day 1), aprepitant (125 mg aprepitant on Day 1, and 80 mg on Days 2 and 3), and olanzapine (5 mg on Days 1–4). The non-olanzapine regimen consisted of the same drug regimen minus olanzapine. The costs and health state outcomes of each treatment were calculated. The model was divided into two phases: the acute phase (Day 1) and the delayed phase (Days 2–5). The clinical outcomes were defined as follows: complete control was defined as no vomiting or retching, no rescue medication use, and no more than mild nausea (0 or 1 on a 4-grade categorical scale). Incomplete control was defined as not achieving complete control.

### Health state outcomes and probabilities

Health state outcomes were evaluated using quality-adjusted life year (QALY). The QALY in each treatment group was integrated according to the probability of the health state in the acute and delayed phases. In Japan, when conducting cost-effectiveness analysis, the use of preference-based measure (PBM) with a value set developed in Japan using time-trade off (TTO) (or mapped onto a TTO score) is recommended as the first choice [19]. The National Institute for Health and Care Excellence (NICE) in the United Kingdom (UK) recommends using the EuroQol 5 Dimension (EQ-5D) [20, 21]. For these reasons, we set the utility value measured by EQ-5D in Japan for our analysis. Utility values of 0.827 for complete control and 0.605 for incomplete control were set. The utility values for complete control and incomplete control were based on Hirose et al., who evaluated the QOL of patients receiving chemotherapy in an outpatient setting using the EQ-5D 5-level (EQ-5D-5L) [22]. Hirose et al. reported a utility value of 0.827 for patients receiving outpatient chemotherapy and a utility value of 0.605 for patients with grade 2 or higher nausea. If drowsiness occurred, the utility value was assumed to decrease by 0.090 based on Hashimoto et al. [23]. Hashimoto et al. investigated the association between sleep disorder and QOL in Japanese patients with type 2 diabetes using EQ-5D-5L. Since no studies assessed the QOL of drowsiness with EQ-5D in cancer patients, we set the disutility due to drowsiness based on Hashimoto et al. They reported that the utility value for patients with daytime sleepiness was 0.73 and that for patients without daytime sleepiness was 0.82. In the J-FORCE study, the probability of drowsiness was reported to be higher for olanzapine, but the probability of daytime sleepiness was significantly higher only on day 1 [8]. Therefore, it was assumed that the utility values were reduced due to drowsiness only on Day 1. The health state probabilities and the probability of drowsiness were based on the J-FORCE study results (Table 1) [8]. The sum of the 5-day QALY was calculated using the following formula:

$$\mathrm{QALY}=\left(\left[\mathrm{PCA}\ast \mathrm{UCC}+\left(1-\mathrm{PCA}\right)\ast \mathrm{UIC}\right)\right]\ast 1\mathrm{d}+\left[\mathrm{PCD}\ast \mathrm{UCC}+\left(1-\mathrm{PCD}\right)\ast \mathrm{UIC}\right]\ast 4\mathrm{d}-\mathrm{PD}\ast \mathrm{DUD}\ast 1\mathrm{d}\Big)/365\mathrm{d}$$

DUD: disutility due to drowsiness; PCA: the probability of complete control in the acute phase; PCD: the probability of complete control in delayed phase; PD: the probability of drowsiness; UCC: utility value of complete control; UIC: utility value of incomplete control; d: Day.

**Table 1:** Utility values for model patient’s outcomes and health state probabilities in the model

Parameters	Base case (Range for one-way sensitivity analysis)	Distribution type for PSA	Reference
Utility weight			
Complete control	0.827 (0.816 ~ 0.837)	Beta (Mean = 0.827, SE = 0.006)	[22]
Incomplete control	0.605 (0.454 ~ 0.756)	Beta (Mean = 0.222, SE = 0.077)	[22]
Disutility due to drowsiness	0.090 (0.050 ~ 0.120)	Beta (Mean = 0.090, SE = 20% of base case)	[23]
Probabilities			
Complete control in acute phase in olanzapine regimen	0.941 (0.911 ~ 0.963)	Beta (α = 333, ß = 21)	[8]
Complete control in acute phase in non-olanzapine regimen	0.880 (0.842 ~ 0.912)	Beta (α = 309, ß = 42)	[8]
Complete control in delayed phase in olanzapine regimen	0.780 (0.733 ~ 0.822)	Beta (α = 276, ß = 78)	[8]
Complete control in delayed phase in non-olanzapine regimen	0.635 (0.583 ~ 0.686)	Beta (α = 223, ß = 128)	[8]
Drowsiness in olanzapine regimen	0.430 (0.380 ~ 0.480)	Beta (α = 153, ß = 202)	[8]
Drowsiness in non-olanzapine regimen	0.330 (0.280 ~ 0.380)	Beta (α = 116, ß = 235)	[8]

*PSA* Probabilistic sensitivity analysis

### Cost

The costs of prophylactic antiemetic therapy and rescue treatments for CINV were included in the model. All costs for drugs in this study were based on the National Health Insurance (NHI) Drug Price Standard listed in 2021 (Table 2). Since generics have been approved for aprepitant and palonosetron, the NHI prices for generics were set. Although generic olanzapine has also been approved, in order to clarify whether the branded type is cost-effective, the base-case analysis was conducted using the branded price, and the analysis was also conducted using the generic price. The rescue treatment cost was set at 833.8 JPY for incomplete control in the acute phase, regardless of the health state of the delayed phase. If there was complete control in the acute phase but incomplete control in the delayed phase, it was set at 286.5 JPY. In the TRIPLE study comparing the efficacy of palonosetron and granisetron [25], Shimizu et al. reported a rescue treatment cost of 833.8 JPY for patients in the palonosetron group who did not achieve complete response (non-CR) in the acute phase and 286.5 JPY for patients with CR in the acute phase but non-CR in the delayed phase [24]. Since the J-FORCE study was conducted in an inpatient setting and the period of analysis in this study was five days, we did not include additional hospitalization costs because we assumed that hospitalization costs would be the same for both groups. Indirect costs were not included because the analysis was performed from the perspective of the Japanese healthcare payer, as described later. The sum of the 5-day cost was calculated using the following formula:

$$\mathrm{Cost}=\mathrm{CAR}+\left(1-\mathrm{PCA}\right)\ast \mathrm{CRA}+\mathrm{PCA}\ast \left(1-\mathrm{PCD}\right)\ast \mathrm{CRD}$$

CAR: Cost of prophylactic antiemetic regimen; CRA: Cost of rescue treatments incomplete control in the acute phase; CRD: Cost of rescue treatments complete control in the acute phase but incomplete control in the delayed phase.

**Table 2:** Costs of drugs and rescue treatment

Study drug costs	Cost (JPY) (Range for one-way sensitivity analysis)	Distribution type for PSA	Reference
Olanzapine 5.0 mg (oral)	150.4 (28.9 ~ 150.4)	Did not vary	NHI price list
Aprepitant 125 mg (oral)	1659.4	Did not vary	NHI price list
Aprepitant 80 mg (oral)	1125.0	Did not vary	NHI price list
Palonosetron 0.75 mg (intravenous)	5349.0	Did not vary	NHI price list
Dexamethasone 4.0 mg (oral)	29.9	Did not vary	NHI price list
Non-olanzapine regimen (APR + PALO + DEX)	9527.5	Did not vary	NHI price list
Olanzapine regimen (OLA + APR + PALO + DEX)	10,129.1 (9643.1 ~ 10,129.1)	Did not vary	NHI price list
Rescue treatments (IC for acute phase)	833.8 (167.1 ~ 1500.5)	Normal (Mean = 833.8, SE = 340.1)	[24]
Rescue treatments (CC for acute phase, IC for delayed phase)	286.5 (97.3 ~ 475.7)	Normal (Mean = 286.5, SE = 96.5)	[24]

Non-olanzapine regimen comprised 12 mg Dexamethasone on Day 1 and 8 mg on Days 2–4, 0.75 mg palonosetron on Day 1, 125 mg aprepitant on Day 1 and 80 mg on Days 2 and 3

*APR* aprepitant, *CC* complete control, *CI* confidence interval, *DEX* dexamethasone, *IC* incomplete control, *JPY* Japanese Yen, *NHI* National Health Insurance, *OLA* olanzapine, *PALO* palonosetron, *PSA* probabilistic sensitivity analysis

### Base-case analysis

The primary outcomes were the expected costs and expected gained QALY. The analysis period was set at five days. No discount was applied since the study lasted for less than a year. The cost-utility analysis was performed from the perspective of the Japanese healthcare payer. Cost-utility analyses were conducted using TreeAge® Pro 2019 (TreeAge Software Inc. Williamstown, MA, USA). The incremental cost-effectiveness ratio (ICER) was calculated. The willingness-to-pay (WTP) threshold was set at 5 million JPY per QALY gained defined by Shiroiwa et al. [26]. The ICER was calculated using the following formula:

$$ICER\;(JPY/QALY)\;=\;(cost\;of\;olanzapine\;regimen-cost\;of\;non-olanzapine\;regimen)/(QALY\;of\;olanzapine\;regimen-QALY\;of\;non-olanzapine\;regimen)$$

In the base-case analysis, ICER was calculated using the branded price of olanzapine. In addition, ICER was also calculated using the generic price of olanzapine.

### One-way sensitivity analysis

A one-way sensitivity analysis was conducted to examine the influence of each parameter on the model. The drug price of olanzapine per 5 mg was varied from 28.9 JPY, the drug price of the generic, to 150.4 JPY, the drug price of the branded. The rescue treatment cost was varied within the 95% confidence interval (CI), calculated based on the cost reported by Shimizu et al. using the following formula assuming a normal distribution:

$$95\%\mathrm{CI}=\mathrm{Mean}\pm 1.96\times \frac{\mathrm{SD}}{\sqrt{\mathrm{n}}}\ \left(\mathrm{SD}:\mathrm{standard}\ \mathrm{deviation};\mathrm{n}:\mathrm{sample}\ \mathrm{size}\right)$$

The health state, drowsiness probabilities, and disutility due to drowsiness varied within the 95%CI [8, 23]. The utility values for complete control and incomplete control were varied within the 95%CI assuming a beta distribution. The 95% CI of the beta distribution was calculated by EZR after the following formula calculated the standard error [27].

$$\mathrm{SE}=\kern0.5em \sqrt{Mean\kern0.5em \left(1- Mean\right)/n}\ \left(\mathrm{SE}:\mathrm{standard}\ \mathrm{error};\mathrm{n}:\mathrm{sample}\ \mathrm{size}\right)$$

EZR (Saitama Medical Center, Jichi Medical University, Saitama, Japan) is a graphical user interface for R (The R Foundation for Statistical Computing, Vienna, Austria). More precisely, it is a modified version of R commander designed to add statistical functions frequently used in biostatistics [28].

The beta distribution was applied because utility values can take values between 0 and 1 (values below zero are possible, but seldom observed) and because it has been widely used in economic evaluation of the distribution of utility values [27]. Threshold analysis was conducted to determine the cost of olanzapine that would make the ICER equivalent to the WTP. In addition, values equal to WTP were calculated for parameters for which ICER exceeded WTP in the one-way sensitivity analysis.

### Probabilistic sensitivity analysis

A probabilistic sensitivity analysis (PSA) was conducted to evaluate the robustness of the base-case analysis. A Monte Carlo simulation was conducted for 10,000 iterations of each comparison. The type of distribution of each parameter for the PSA is shown in Table 2. A beta distribution was applied to the utility values and health state probabilities. A beta distribution is a type of distribution that takes values between 0 and 1, and its mean and SD are expressed by the following formula:

$$\mathrm{Mean}=\kern0.5em \frac{\alpha }{\alpha +\beta },\mathrm{SD}=\kern0.5em \sqrt{\frac{\alpha \beta}{{\left(\alpha +\beta \right)}^2\left(\alpha +\beta +1\right)}}\left(\alpha :\mathrm{number}\ \mathrm{of}\ \mathrm{events};\beta :\mathrm{number}\ \mathrm{of}\ \mathrm{non}-\mathrm{events}\right)$$

For utility values, SE values were set to the SD of the beta distribution to vary the population means. The SE of disutility due to drowsiness was assumed to be 20% of the base case because the SD and SE were not reported.

## Results

### Base-case analysis

The cost was 10,238 JPY in the olanzapine regimen and 9719 JPY in the non-olanzapine regimen. The QALY gained were 0.01065 QALYs in the olanzapine regimen and 0.01029 QALYs in the non-olanzapine regimen. The incremental cost of the olanzapine regimen relative to the non-olanzapine regimen was 519 JPY, and the incremental QALYs were 0.00036 QALY, resulting in an ICER of 1,428,675 JPY per QALY gained. This value was below the WTP, and according to the base case analysis, the olanzapine regimen was more cost-effective than the non-olanzapine regimen. When using the generic olanzapine, the cost in the olanzapine regimen and an ICER were decreased to 9752 JPY, and 90,059 JPY per QALY gained, respectively.

### One-way sensitivity analysis

**Fig. 2:**
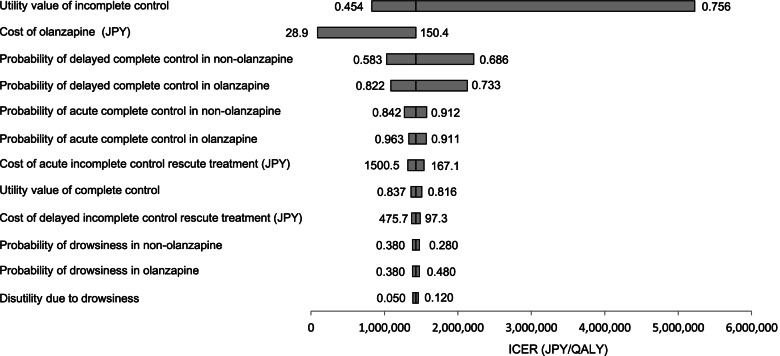
The result of one-way sensitivity analysis. One-way sensitivity analysis represents the influence of each parameter on the model. ICER, incremental cost-effectiveness ratio; JPY: Japanese Yen; QALY, quality-adjusted life-year

A tornado diagram based on the one-way sensitivity analysis is shown in Fig. 2. It is arranged in order of the degree of influence on ICER. The most influential parameter on ICER was the utility value of incomplete control, followed by the utility value of complete control, the probability of delayed complete control in non-olanzapine, and the probability of delayed complete control in olanzapine (Fig. 2). The drug price of olanzapine per 5 mg with ICER equal to WTP was 475 JPY. The parameter that exceeded WTP over the varied range was the utility value of incomplete control, with ICER and WTP equal at 0.754.

### Probabilistic sensitivity analysis

**Fig. 3:**
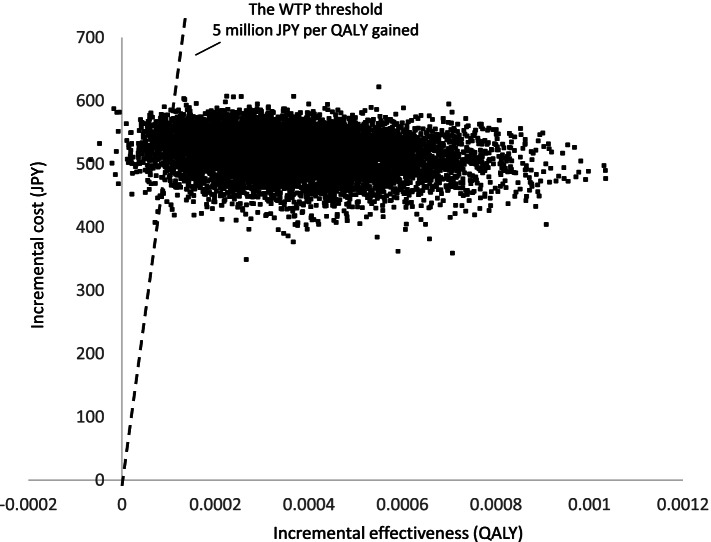
Scatter plot showing results of probabilistic sensitivity analysis. In the Scatter plot, a point that exists to the lower right of the WTP threshold and has positive incremental effectiveness is cost-effective. ICER, incremental cost-effectiveness ratio; JPY, Japanese Yen, QALY, quality-adjusted life year; WTP, willingness-to-pay

**Fig. 4:**
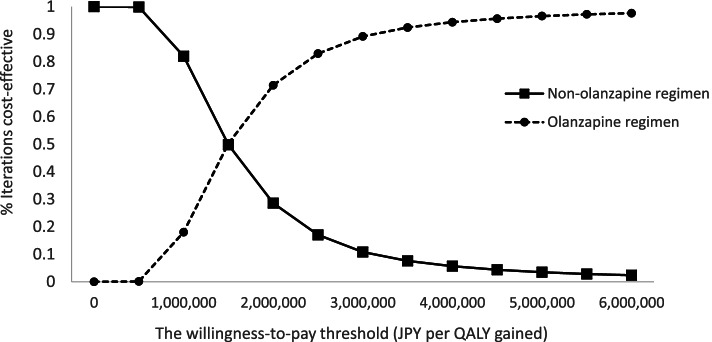
Cost-effectiveness acceptability curve. JPY, Japanese Yen, QALY, quality-adjusted life year; WTP, willingness-to-pay

The PSA results are shown in a scatter plot (Fig. 3). The PSA revealed the probability that the ICER was below the WTP, and the incremental QALYs was positive was 96.2%. Based on the cost-acceptability curve, this probability was almost equal for the olanzapine and the non-olanzapine regimen when the WTP threshold was set at 1.5 million JPY (Fig. 4).

## Discussion

To the best of our knowledge, this was the first report to evaluate the cost-effectiveness of olanzapine in a four-drug antiemetic regimen in Japan. Since cost-effectiveness was shown for both branded and generic, olanzapine in a four-drug antiemetic regimen is considered cost-effective. The most significant difference from the previous cost-effectiveness analysis of antiemetic drugs is that the utility value is based on the EQ-5D of the Japanese population. For this reason, the utility value for incomplete control in the base-case analysis was set at 0.605, which is very different from the utility values set by many studies in the past (0.20 or 0.27) [12–17]. The utility value differs significantly depending on the country, disease, and scale used for measurement. The value of 0.20 and 0.27 values are based on the results of the visual analog scale (VAS) evaluation of nausea alone and differ significantly from EQ-5D, Health Utilities Index-8 (HUI-8), and Short form 6 dimension (SF-6D), which evaluate QOL comprehensively using multiple dimensions. Therefore, the utility value of incomplete response may be underestimated, and the incremental QALYs may be overestimated when analyzed using the utility value evaluated by VAS. Chanthawong et al. reported that compared to the doublet antiemetic regimen (dexamethasone plus first-generation 5-HT_3_ receptor antagonist), the addition of olanzapine resulted in incremental QALY of 0.0022–0.0026 [17]. At the same time, they reported that in Singapore, switching from aprepitant to olanzapine in a triplet antiemetic regimen (dexamethasone, first-generation 5-HT_3_ receptor antagonist, and aprepitant) resulted in incremental QALY of 0.0005. In Japan, there have been several reports examining the cost-effectiveness of aprepitant and palonosetron [13, 15, 16]. Kashiwa et al. reported the cost-effectiveness of palonosetron in cisplatin-containing HEC regimens based on the TRIPLE study results conducted in Japan [14, 25]; the incremental effect of palonosetron was 0.000645 QALYs. Tsukiyama et al. reported that the incremental effect of aprepitant was 0.016 QALYs [16]. Compared to these previous studies, the incremental effect of our study was much smaller at 0.00036 QALYs. This may be the difference in the utility value of incomplete control. NICE in the UK recommends using EQ-5D to measure utility values during cost-effectiveness analysis because it is a standardized measure validated in many patients. The Central Social Insurance Medical Council of Japan guidelines state that cost-effectiveness analysis can only be used with patient PBM such as EQ-5D, HUI, and SF-6D. In addition, EQ-5D is one currently available measure for which a scoring algorithm has been developed in Japan. For these reasons, it is reasonable that we used the utility value calculated by EQ-5D for Japanese subjects in this study.

Chow et al. reported that the olanzapine regimen was dominant to the non-olanzapine regimen based on US drug prices [18]. Chow et al. set the cost of uncontrolled CINV at 1883 USD (147,670 JPY) based on the report of Shih et al. conducted in the US from 1997 to 2002 [29], which differs significantly from the cost of rescue treatment of our study. The report by Shih et al. calculates the additional cost per month based on the 1997–2002 Medstat MarketScan Health and Productivity Management database, a large, nationwide, employment-based database collected from approximately 45 large employers in the US and over 100 health insurance payers. Although the details of the additional costs are not described, the reasons for the difference may be that medical costs differ significantly between Japan and the US, and the data were obtained before aprepitant and palonosetron were launched in the US. In Japan, Hamada et al. reported that patients who experienced CINV had an additional 170 USD per course compared to those who did not [30]. However, we did not cite the additional costs reported by Hamada et al. in this study because of the following reasons: first, their data were from 2005 to 2007 before aprepitant and palonosetron were launched in Japan; second, most of the additional costs are additional drug medication costs, and the antiemetic drugs used have changed significantly since that time; third, the cost was per course of cisplatin, which differs from the 5 days of our observation period. Although we set the rescue treatment cost based on the report by Shimizu et al. [24], since the TRIPLE study was conducted in 2011–2012 [25], we expect that the rescue treatment cost of Shimizu et al.’s report will be lower now that generics of granisetron, palonosetron, olanzapine, and aprepitant are available. However, the effect of the rescue treatment cost was small in the one-way sensitivity analysis, and even if the rescue treatment cost was smaller, the ICER was below the WTP threshold, suggesting that the rescue treatment cost had little effect on the results.

Chow et al. and Chanthawong et al. did not include adverse events of olanzapine in their model, whereas we accounted for disutility due to drowsiness [17, 18]; nevertheless, the result was similar. The disutility due to drowsiness in this study was based on QOL values for Japanese patients with type 2 diabetes [23], not cancer patients. However, since the effects of drowsiness and disutility due to drowsiness were small in the one-way sensitivity analysis, the effects of drowsiness and disutility on the results of this study were also small. Since the disutility value of drowsiness, which appeared most frequently, had little effect on the results, the impact of adverse effects other than drowsiness on the results were considered to be small. The use of antiemetics can lead to hospitalization for drug-induced adverse events such as paralytic ileus. In the J-FORCE study, one case of Grade 3 constipation was reported [8]. However, since the probability is minimal (1/355) and our study was conducted in an inpatient setting, the effect of not including additional hospitalization costs is likely to be small.

This study had some limitations. First, the utility values could not be used directly measured in the J-FORCE study. In addition, the J-FORCE study was conducted in an inpatient setting, whereas the Hirose et al. report we cited was in an outpatient setting [8, 22]. However, we believe that we could obtain reliable results because the utility values were set based on EQ-5D measured in Japan as recommended by domestic and international guidelines, not based on overseas VAS as in previous reports [12–17]. In addition, more than 95% of the J-FORCE study patients had an ECOG-PS of 0 or 1 and were in good performance status. The reason the J-FORCE study was conducted in an inpatient setting may have been to evaluate efficacy and safety. For these reasons, it is believed that there is a certain validity in using utility values for patients in the outpatient setting in this study’s analysis. However, based on the findings of this study that utility values have a significant impact on ICER, it is clearly desirable to set utility values that are directly evaluated in clinical trials. Second, the range of values for complete response in the sensitivity analysis may be narrow. The reason for the narrow range is that the Hirose et al. study was a large investigation of 1008 patients with 4695 QOL surveys (40 surveys for patients with grade 2 or higher nausea), and the 95% CIs were calculated with high precision [22]. However, utility values have been reported to vary among cancer types and patient ethnicities (e.g., 0.62 for Chinese colorectal cancer patients and 0.90 for UK prostate cancer patients [31, 32]), and if only a single study is included, the uncertainty may be underestimated. However, it is the difference between the utility value of complete and incomplete response that affects the ICER, and we had set the utility value for incomplete response over a sufficiently wide range. The PSA showed that despite the wide range of utility value of incomplete response, the probability of ICER being below WTP was high at 96.2%. Therefore, we believe that the range of variation in the baseline utility values has a small impact on the study’s conclusions. Third, the study was conducted in an inpatient setting. However, if the study is conducted under outpatient setting, additional hospital visits and hospitalization costs may be incurred when CINV appears. In fact, taking these effects into account, Tsukiyama et al. reported that aprepitant was more cost-effective in the outpatient setting than in the inpatient setting [16]. Furthermore, if the opportunity for additional hospital visits can be reduced by control of CINV, there will be advantages in terms of indirect costs, such as loss of productivity due to missed work and reduced hospital visit costs. From these perspectives, olanzapine, which showed excellent cost-effectiveness in the inpatient condition, is also considered cost-effective in the outpatient setting. Fourth, the analysis period was short, at only five days; an even longer period could be considered, such as one year. The rationale for setting the observation period to five days is as follows: first, prophylactic antiemetic therapy has not been proven to improve long-term QOL or prolong survival. Second, the efficacy of olanzapine in clinical trials, including the J-FORCE study, was evaluated for only five days in the first course. If CIVN prophylaxis improves long-term outcomes, the gained QALY will be even greater than this study; however, the conclusion that olanzapine is highly cost-effective remains unchanged. In the future, we hope that the impact of CINV on long-term outcomes will be clarified.

## Conclusions

In conclusion, olanzapine was highly cost-effective in highly emetogenic cisplatin-containing risk regimens. Therefore, the use of a four-drug regimen, including olanzapine, was recommended in terms of cost-effectiveness.

## Data Availability

The datasets used and/or analyzed during the current study are available from the corresponding author on reasonable request.

## Abbreviations

ASCO: American Society of Clinical Oncology
CI: Confidence interval
CAR: Cost of prophylactic antiemetic regimen
CINV: Chemotherapy-induced nausea and vomiting
CR: Complete response
CRA: Cost of rescue treatments incomplete control in the acute phase
CRD: Cost of rescue treatments complete control in the acute phase but incomplete control in the delayed phase
DUD: Disutility due to drowsiness
ECOG-PS: Eastern Cooperative Oncology Group performance status
EQ-5D-5L: EuroQOL 5 Dimension 5-level
ESMO: European Society of Medical Oncology
HEC: Highly emetogenic chemotherapy
5-HT: 5-hydroxytryptamine
HUI-8: Health Utilities Index-8
ICER: Incremental cost-effectiveness ratio
JPY: Japanese yen
MASCC: Multinational Association of Supportive Care in Cancer
NCCN: National Comprehensive Cancer Network
NICE: National Institute for Health and Care Excellence
NHI: National Health Insurance
NK1: Neurokinin-1
PBM: Preference-based measure
PCA: The probability of complete control in the acute phase
PCD: The probability of complete control in the delayed phase
PD: The probability of drowsiness
PSA: Probabilistic sensitivity analysis
QALY: Quality-adjusted life year
QOL: Quality of life
SD: Standard deviation
SF-6D: Short form 6 dimension
TTO: Time-trade off
UCC: Utility value of complete control
UIC: Utility value of incomplete control
UK: United Kingdom
USD: United States dollars
VAS: Visual analogue scale
WTP: Willingness-to-pay
